# *Waiting for Godot* – Epilogue

**DOI:** 10.1017/dmp.2020.307

**Published:** 2020-12

**Authors:** James J. James

As noted in a prior editorial, *Waiting for Godot* was a 2-act play by Stephen Beckett, which was used as a metaphor for our response to coronavirus disease (COVID-19).^1^ The original play represented what has become known as the *Theatre of the Absurd*, the hallmarks of which include meaningless dialogue, plots without logical development and dystopic realities – all characteristics of many of our efforts to control the COVID-19 pandemic. Herein, with apologies to Beckett, an epilogue is presented, which further elaborates on our ongoing pandemic response that is primarily based on observation and logic, as opposed to assumption-based predictive modeling and unproven hypotheses. The overall intent is to better understand where we are now, how we got here, and, most importantly, begin the process of better preparing for the next pandemic. We can no longer await the arrival of what might never come but must instead consider actions that will move us forward in terms of recovering from the current pandemic while preparing to better respond in the future. As a device to advance the conversation, a series of challenging questions will be asked.

On March 11 2020, the World Health Organization (WHO) declared COVID-19 a pandemic, which immediately raised fear levels to a state of panic around the world, precipitated a loss of 5000 points in the Dow Jones Industrial Average, and resulted in severe global socioeconomic damage. This declaration was based on 118 000 reported cases with 4300 deaths in 114 countries.^2^ Although this action had a global life-changing impact, few have asked, “Was the declaration justified?” In attempting to provide an answer to this question, we need to first point out that, for other disasters such as earthquakes and hurricanes, we have scales to help assess the intensity of the individual events and their consequences. For pandemics, we have no such measures, although we have multiple scalar parameters available, such as morbidity, mortality, and geographic spread that directly influence health impacts. Given the extreme consequences of a pandemic declaration, the availability of a Pandemic Scale would permit a more gradated response as opposed to an all-or-none dichotomy. As a rudimentary example of such a scale, we can look to historic pandemics and estimate their health impacts on global populations. Although a daunting exercise because of limited and non-standardized data, some generally accepted approximations, based on estimated overall mortality, are available.^3^ Using best-estimate global population figures in conjunction with these data, we can infer deaths per 10 000 population for representative pandemics (Table 1).

**TABLE 1:** Historic Pandemic Mortality (PM) in Deaths Per 10 000 Per Year (*Note*. for Justinian and Black Death, the PMs are adjusted to show yearly averages.)

Pandemic	Years	Agent	Deaths	Population	PM (est./yr)
Justinian	541-542	Yersinia pestis	40 Million	200 Million	1000
Black Death	1347-1351	Yersinia pestis	200 Million	400 Million	1250
Third plague	1885	Yersinia pestis	12 Million*	600 Million	200
Russian flu	1889-1890	Influenza	1 Million	1 Billion	10
Spanish flu	1918	Influenza	45 Million	1.8 Billion	250
Asian flu	1957-1958	Influenza	1.1 Million	3 Billion	3.6
Swine flu	2009-2010	Influenza	200 000	6 Billion	0.3
COVID-19	March 11, 2020	SARS-CoV-2	4330	7.8 Billion**	0.005
COVID-19	March 17, 2020	SARS-CoV-2	800 000	7.8 Billion**	1.0

*Notes.* *Limited to China and India; **Ongoing.

The PMs calculated in this exercise are based on mortality alone and do not address overall morbidity or geographic spread, which are certainly important elements in defining overall pandemic severity. However, at the same time, mortality is and has been the single most important factor in measuring overall pandemic impact and has the advantage of having a finite measurable outcome. Using this measure in the context of Table 1 would certainly call into question declaring COVID-19 a pandemic, whereas the global spread would certainly justify that decision. To reconcile this paradox, which will certainly present itself again in the future, a pandemic index, akin to a Richter Scale, needs to be developed under the auspices of the WHO in collaboration with global health authorities. This would allow a much more nuanced declaration in the future and could go a long way to controlling the fear and panic that have driven our response(s) to COVID-19. An event with the mortality of the Black Death could well be a 10 with the swine flu a possible 1 and COVID-19, using today’s mortality data, somewhere around a 2 or 3 but adjustable with the evolution of the pandemic.

As noted previously, mortality is the generally accepted indicator for the severity of a pandemic. For COVID-19, however, we continue to measure the impact in terms of a positive lab test, which we use to define a “case” even though many positives are asymptomatic, which is a contradiction in terms. Without dwelling on the many operative reasons for this, the overarching outcome is fear-based, public health messaging relying on large numbers, often presented out of context, without a denominator, and amplified in the traditional and social media.^4^ This brings up the second question, “What is the usefulness of the COVID-19 PCR test?” As with all things related to COVID-19, not only are there many conflicting views, but also each can find support somewhere among the many thousands of articles published in the medical and public health literature. There is little to debate as to the test’s usefulness as a diagnostic tool for those exhibiting symptoms, and this will become increasingly important as we approach the flu season. The real issue relates to its usefulness as a public health measure to contain and mitigate the spread of the virus. Given that we are currently reporting some 50 000 new positives each day from across 99% of US counties, we do need to consider that little can be done to limit the pandemic’s geographic spread.

The second public health application of the polymerase chain reaction (PCR) test is to enable contact tracing, a tried and true tool to identify those potentially infected and limit additional exposure to the pathogen. There are many issues to consider here, not the least of which is the enormous human and laboratory capacity needed to adequately perform contact tracing on over 350 000 new cases each week. These difficulties are further amplified by the reluctance of many to identify contacts, resistance to self-quarantine at home for 2 weeks, and potential increased exposure to other household members. Any problems with test characteristics and processing aside, we need to keep in mind 2 additional thoughts: that the PCR is a point-in-time test, and a negative today could be a positive tomorrow and vice versa, and an infected individual can test negative while a recovered individual can test positive. Finally, as 80 to 85% of symptomatic positives experience a mild to moderate illness, the number of PCR positives is a poor measure of the health impacts of COVID-19. Unfortunately, there are primary beneficiaries, in terms of ratings and advertising dollars, of selectively reporting and sensationalizing new positive tests per day and propagating the attendant fear, and these are our media outlets.^5^

This introduces the third question, “Does the risk justify the level of fear?” To address this, we need to look at why we fear COVID-19. There are 2 salient reasons that can be identified. First, as any student of the psychology of fear knows, we are most afraid of the unknown, especially the invisible, and new infectious diseases are prime examples of such threats. Hopefully, we can partially allay that fear in that we have experienced over 22 million cases, and now have a significant understanding of the epidemiology of COVID-19, especially with regard to who is at greatest risk and how to better protect them. The second reason for the high level of fear is that, for those who do have a serious case, it is a nasty and potentially fatal disease and, in densely populated cities, the numbers of the critically ill can overwhelm medical facilities and staff. To reduce this second reason for fear, we need to encourage individuals to better assess their individual risk and that of those close to them under different circumstances and conditions through sound health communication. Most importantly, messages need to stress that COVID-19 is here and it is not going away anytime soon. It joins a host of other afflictions and exposures that carry a certain degree of risk that we have learned to accommodate and live with, and, as with other risks, there are protective measures we can take to reduce the risk of COVID-19. As of today, the overall attack rate for the United States, as measured by a positive PCR, is less than 2% and the risk of a fatal outcome is 0.05% of which 80% occur in those over age 65. These risks are further lessened in terms of the epidemiologic curve, which has trended steadily down over the past month, and daily positives are lower by almost 30% with a significant drop in average age.^6^

The above is in no way intended to downplay the very real significant medical impact of COVID-19 but to put it in a more objective context, with the aim of reducing the fear level and lessening the significant collateral damage to our socioeconomic well-being, resulting from our more extreme interventions, such as lockdowns and school closures.^7^ These become evermore damaging as they are extended with no real end point in sight awaiting the effective vaccine that may or may not arrive. Which brings up the final question, “What is the role of herd immunity (HI)?” The discussion on HI and COVID-19 has been controversial from the beginning with too many equating it to purposefully exposing individuals to a potentially lethal disease which is simply not the case. HI is not an intervention and it evolves naturally as the number of immune-protected individuals in the population increases. The real issue is the level of immunes that must be achieved to subdue transmission. The immunes represent the recovered plus the vaccinated (when available) when all population members are equally susceptible, which is far from the case with COVID-19. With this disease, we must also adjust for a degree of relative immunity, which is inversely proportionate to age and varies with other factors. Accounting for this heterogeneity suggests that an effective HI may be 40% or less as opposed to early estimates of 70–80% which assumed population homogeneity.^8^ Given the lowered HI targets plus the findings from several serologic surveys demonstrating prevalence levels of 20% and above, an effective level of HI may have already been achieved in New York City (NYC) and other large cities. In fact, it is not inconceivable that the downward trend in the epidemic curve reflects a partial HI effect and each new test positive contributes to the immune protected in the population and decreases the risk of transmission among the remaining at-risk individuals.

Considering the factors noted above, most adults can be depended upon to take the known precautions that decrease their risk and, just as importantly, can assume the degree of risk with which they are comfortable. Unfortunately, our children cannot make these assessments on their own and interventions are decided for them. As a result, widespread school closures have been enforced with up to 55 million K–12 grade children made to suffer educational and social deprivation, and, in many cases, abuse as well as psychological and emotional distress, nutritional diminution, and an alarming interruption in child preventive health services to include basic immunizations; ironically, this is the very age group at extremely low risk for serious COVID-19 outcomes. Yet, we face another semester when many major school systems will again be closed purportedly based on the fear that a student may be exposed, test positive and become ill, or expose others outside of school. Rather than go into lengthy counter arguments to these points, I would offer the following quote by NYC Health Commissioner Royal S. Copeland on his decision to keep the city schools open during the 1918 flu pandemic:

“New York is a great cosmopolitan city and in some homes there is careless disregard for modern sanitation… In schools the children are under the constant guardianship of the medical inspectors. This work is part of our system of disease control. If the schools were closed at least 1,000,000 would be sent to their homes and become 1,000,000 possibilities for the disease. Furthermore, there would be nobody to take special notice of their condition.”^9^

Commissioner Copeland was highly regarded for his effective management of the 1918 pandemic, and his thought processes ring true today, most especially regarding our most disadvantaged children.

In closing, I would make a final observation in our epilogue. Possibly, Godot has been in plain sight, but we have been ignoring him. The hallmark of the 1918 NYC response is that health decisions rested in large part with the public health authorities and not with elected officials currying political favor. Consequently, the primary goal was the overall public good and not advancing a political agenda as is too often the case today.
